# Crystal structure of 6,7-di­chloro-4-oxo-4*H*-chromene-3-carbaldehyde

**DOI:** 10.1107/S2056989015014644

**Published:** 2015-08-12

**Authors:** Yoshinobu Ishikawa

**Affiliations:** a School of Pharmaceutical Sciences, University of Shizuoka, 52-1 Yada, Suruga-ku, Shizuoka 422-8526, Japan

**Keywords:** crystal structure, chromone, hydrogen bonding, halogen–halogen contact, stacking inter­action

## Abstract

In the title compound, C_10_H_4_Cl_2_O_3_, a dichlorinated 3-formyl­chromone, the non-H atoms of the 4*H*-chromene ring are essentially coplanar (r.m.s. = 0.0188 Å), with the largest deviation from the least-squares plane [0.043 (2) Å] being for the pyran C=O C atom. The α,β-unsaturated carbonyl O atom deviates from the least-square plane by 0.124 (2) Å. The dihedral angle between the chromone and formyl least-square planes is 6.76 (3)°. In the crystal, mol­ecules are linked through C—H⋯O hydrogen bonds between the translation-symmetry and inversion-symmetry equivalents to form tetrads, which are further assembled by stacking inter­actions [centroid–centroid distance between the benzene rings = 3.769 (2) Å]. van der Waals contacts are found between the Cl atoms at the 6-position and the Cl atoms at 7-position of the glide-reflection-symmetry equivalents [Cl⋯Cl = 3.4785 (16) Å, C—Cl⋯Cl = 160.23 (7)° and Cl⋯Cl—C = 122.59 (7)°].

## Related literature   

For related structures, see: Ishikawa & Motohashi (2013 ▸); Ishikawa (2014*a* ▸,*b* ▸, 2015 ▸). For halogen bonding and halogen⋯halogen interactions, see: Auffinger *et al.* (2004 ▸); Metrangolo *et al.* (2005 ▸); Metrangolo & Resnati (2014 ▸); Mukherjee & Desiraju (2014 ▸); Wilcken *et al.* (2013 ▸); Sirimulla *et al.* (2013 ▸); Persch *et al.* (2015 ▸).
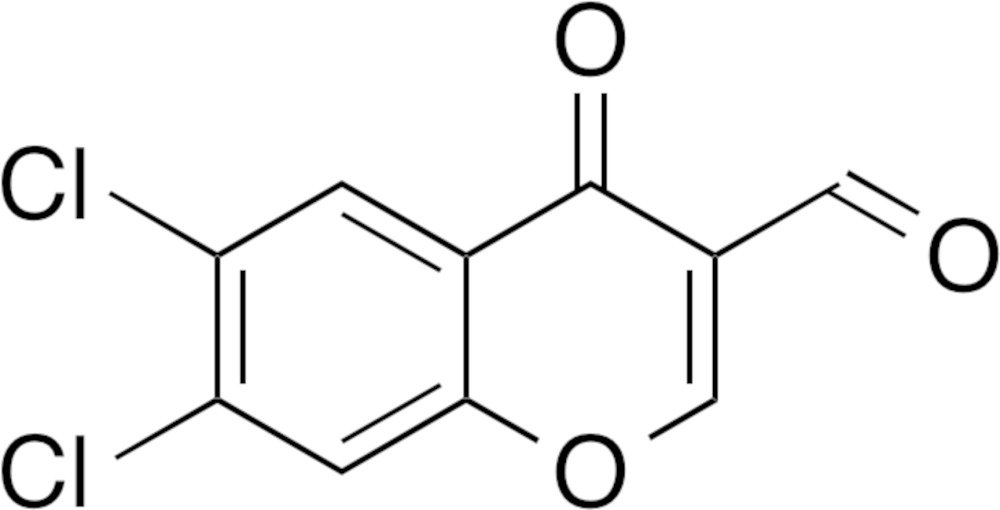


## Experimental   

### Crystal data  

C_10_H_4_Cl_2_O_3_
*M* *_r_* = 243.05
Monoclinic, 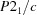

*a* = 3.7695 (13) Å
*b* = 6.1465 (16) Å
*c* = 39.431 (13) Å
β = 90.72 (3)°
*V* = 913.5 (5) Å^3^
*Z* = 4
Mo *K*α radiation
μ = 0.69 mm^−1^
*T* = 140 K
0.30 × 0.25 × 0.10 mm

### Data collection  

Rigaku AFC–7R diffractometer
Absorption correction: ψ scan (North et al., 1968 ▸) *T*_min_ = 0.574, *T*_max_ = 0.934
5075 measured reflections
2089 independent reflections
1747 reflections with *F* ^2^ > 2.0σ(*F* ^2^)
*R*_int_ = 0.052
3 standard reflections every 150 reflections intensity decay: 0.6%

### Refinement  

*R*[*F* ^2^ > 2σ(*F* ^2^)] = 0.034
*wR*(*F* ^2^) = 0.098
*S* = 1.04
2089 reflections
136 parameters
H-atom parameters constrained
Δρ_max_ = 0.29 e Å^−3^
Δρ_min_ = −0.36 e Å^−3^

### 

Data collection: *WinAFC Diffractometer Control Software* (Rigaku, 1999 ▸); cell refinement: *WinAFC Diffractometer Control Software*; data reduction: *WinAFC Diffractometer Control Software*; program(s) used to solve structure: *SIR2011* (Burla *et al.*, 2012 ▸); program(s) used to refine structure: *SHELXL2014* (Sheldrick, 2015 ▸); molecular graphics: *CrystalStructure* (Rigaku, 2015 ▸); software used to prepare material for publication: *CrystalStructure*.

## Supplementary Material

Crystal structure: contains datablock(s) global, I. DOI: 10.1107/S2056989015014644/zl2636sup1.cif

Structure factors: contains datablock(s) I. DOI: 10.1107/S2056989015014644/zl2636Isup2.hkl

**Fig. 1.**
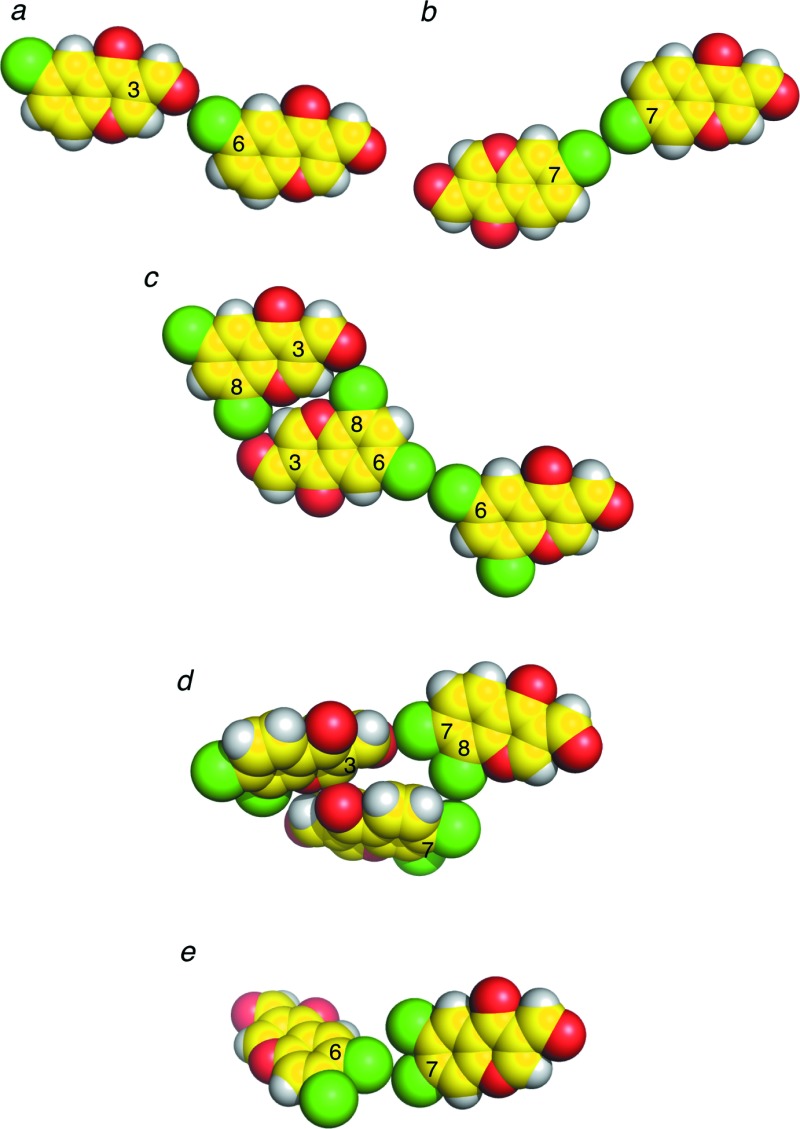
Sphere models of the crystal structures of (a) 6-chloro-4-oxo-4H-chromene-3-carbaldehyde (Ishikawa, 2014a), (b) 7-chloro-4-oxo-4H-chromene-3-carbaldehyde (Ishikawa, 2014b), (c) 6,8-dichloro-4-oxochromene-3-carbaldehyde (Ishikawa & Motohashi, 2013), (d) 7,8-dichloro-4-oxo-4H-chromene-3-carbaldehyde (Ishikawa, 2015) and (e) the title compound (this work).

Supporting information file. DOI: 10.1107/S2056989015014644/zl2636Isup3.cml

a H a b H b c d H e . DOI: 10.1107/S2056989015014644/zl2636fig1.tif
Sphere models of the crystal structures of (*a*) 6-chloro-4-oxo-4*H*-chromene-3-carbaldehyde (Ishikawa, 2014*a*), (*b*) 7-chloro-4-oxo-4*H*-chromene-3-carbaldehyde (Ishikawa, 2014*b*), (*c*) 6,8-di­chloro-4-oxochromene-3-carbaldehyde (Ishikawa & Motohashi, 2013), (*d*) 7,8-di­chloro-4-oxo-4*H*-chromene-3-carbaldehyde (Ishikawa, 2015) and (*e*) the title compound (this work).

**Fig. 2.**
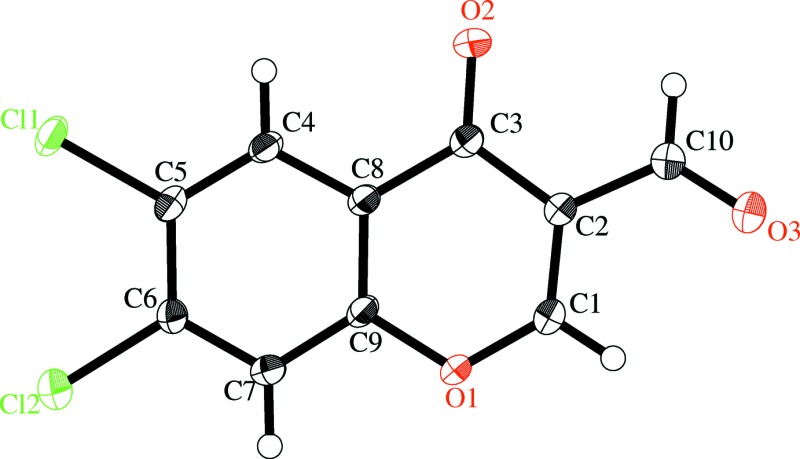
The molecular structure of the title compound with displacement ellipsoids drawn at the 50% probability level. Hydrogen atoms are shown as small spheres of arbitrary radius.

. DOI: 10.1107/S2056989015014644/zl2636fig2.tif
The mol­ecular structure of the title compound with displacement ellipsoids drawn at the 50% probability level. Hydrogen atoms are shown as small spheres of arbitrary radius.

**Fig. 3.**
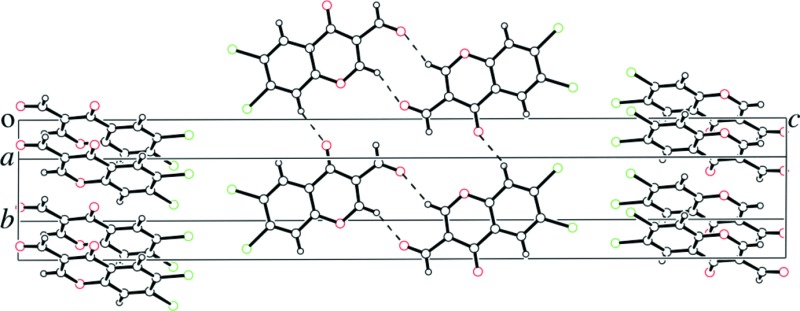
A packing view of the title compound. C–H···O hydrogen bonds are represented by dashed lines.

. DOI: 10.1107/S2056989015014644/zl2636fig3.tif
A packing view of the title compound. C–H⋯O hydrogen bonds are represented by dashed lines.

CCDC reference: 1416757

Additional supporting information:  crystallographic information; 3D view; checkCIF report

## Figures and Tables

**Table 1:** Hydrogen-bond geometry (, )

*D*H*A*	*D*H	H*A*	*D* *A*	*D*H*A*
C1H1O3^i^	0.95	2.34	3.187(3)	148(1)
C7H3O2^ii^	0.95	2.26	3.129(2)	151(1)

Symmetry codes: (i) 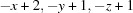; (ii) 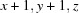.

## supplementary crystallographic information

### S1. Comment

Halogen bonding is an electrostatic interaction between an electrophilic region of a halogen atom and a nucleophilic region of an atom, and has attracted much attention in medicinal chemistry, chemical biology, supramolecular chemistry and crystal engineering (Auffinger *et al.*, 2004, Metrangolo *et al.*, 2005, Wilcken *et al.*, 2013, Sirimulla *et al.*, 2013, Mukherjee & Desiraju, 2014, Metrangolo & Resnati, 2014, Persch *et al.*, 2015). This is characterized by a short contact between the two atoms.

I have reported the crystal structures of chlorinated 3-formylchromones 6-chloro-4-oxo-4*H*-chromene-3-carbaldehyde (Ishikawa, 2014*a*), 7-chloro-4-oxo-4*H*-chromene-3-carbaldehyde (Ishikawa, 2014*b*), 6,8-dichloro-4-oxochromene-3-carbaldehyde (Ishikawa & Motohashi, 2013) and 7,8-dichloro-4-oxo-4*H*-chromene-3-carbaldehyde (Ishikawa, 2015). As for the monochlorinated 3-formylchromones, van der Waals contacts are observed between the formyl oxygen atom and the chlorine atom at 6-position in 6-chloro-4-oxo-4*H*-chromene-3-carbaldehyde (Fig. 1*a*), and between the chlorine atoms at 7-position in 7-chloro-4-oxo-4*H*-chromene-3-carbaldehyde (Fig. 1*b*). On the other hand, as for the dichlorinated 3-formylchromones, halogen bonds are observed between the formyl oxygen atom and the chlorine atom at 8-position in 6,8-dichloro-4-oxochromene-3-carbaldehyde (Fig. 1*c*), and between the formyl oxygen atom and the chlorine atom at 7-position in 7,8-dichloro-4-oxo-4*H*-chromene-3-carbaldehyde (Fig. 1*d*). As part of my investigation into these types of chemical bonding, I herein report the crystal structure of a dichlorinated 3-formylchromone 6,7-dichloro-4-oxo-4*H*-chromene-3-carbaldehyde. The main objective of this study is to reveal the interaction modes of the chlorine substituents of the title compound in the solid state.

The mean deviation of the least-square plane for the non-hydrogen atoms of the 4*H*-chromene ring is 0.0188 Å, and the largest deviation is 0.043 (2) Å for the C3 atom (Fig. 2). The α,β-unsaturated carbonyl O2 atom deviates from the least-square plane by 0.124 (2) Å. The dihedral angle between the chromene least-square plane and the formyl C2–C10–O3 plane is 6.76 (3)°.

In the crystal, the molecules are linked through C–H···O hydrogen bonds between the translation-symmetry^i^ and inversion-symmetry equivalents^ii,iii^ to form tetrads [i: *x* – 1, *y* + 1, *z*, ii: –*x* + 1, –*y*, –*z* + 1, iii: –*x* + 2, –*y* + 1, –*z* + 1], which are further assembled by stacking interactions [centroid–centroid distance between the benzene rings of the 4*H*-chromene units = 3.769 (2) Å], as shown in Fig. 3.

Van der Waals contacts are found between the chlorine atoms at 6-position and the chlorine atoms at 7-position of the glide-reflection-symmetry equivalents^iv^ [Cl1···Cl2^iv^ = 3.4785 (16) Å, C5–Cl1···Cl2^iv^ = 160.23 (7)°, Cl1···Cl2^iv^–C6^iv^ = 122.59 (7)°, iv: –*x* + 1, *y* – 1/2, –*z* + 1/2], as shown in Fig. 1*e*. Thus, short contacts are observed for the chlorine atoms in the title compound. The interaction modes of the chlorine atoms in these dichlorinated 3-formylchromones might depend on how strongly the chlorine atoms interact with the oxygen and other vicinal chlorine atoms intramolecularly. These findings could be helpful to rational drug design considering halogen bonding.

### S2. Experimental

To a solution of 4',5'-dichloro-2'-hydroxyacetophenone (4.8 mmol) in *N*,*N*-dimethylformamide (15 ml) was added dropwise POCl_3_ (12.0 mmol) at 0 °C. After the mixture was stirred for 14 h at room temperature, water (50 ml) was added. The precipitates were collected, washed with water and dried *in vacuo* (yield: 65%). ^1^H NMR (400 MHz, CDCl_3_): *δ* = 7.71 (s, 1H), 8.37 (s, 1H), 8.52 (s, 1H), 10.35 (s, 1H). Single crystals suitable for X-ray diffraction were obtained from a 1,2-dichloroethane solution of the title compound at room temperature.

### S3. Refinement

The C(*sp*^2^)-bound hydrogen atoms were placed in geometrical positions [C–H 0.95 Å, *U*_iso_(H) = 1.2*U*_eq_(C)], and refined using a riding model. One reflection (­–3 0 2) was omitted because of systematic error.

### Crystal data

**Table:**

C_10_H_4_Cl_2_O_3_	*F*(000) = 488.00
*M**_r_* = 243.05	*D*_x_ = 1.767 Mg m^−^^3^
Monoclinic, *P*2_1_/*c*	Mo *K*α radiation, λ = 0.71069 Å
*a* = 3.7695 (13) Å	Cell parameters from 25 reflections
*b* = 6.1465 (16) Å	θ = 15.2–17.2°
*c* = 39.431 (13) Å	µ = 0.69 mm^−^^1^
β = 90.72 (3)°	*T* = 140 K
*V* = 913.5 (5) Å^3^	Plate, yellow
*Z* = 4	0.30 × 0.25 × 0.10 mm

### Data collection

**Table:**

Rigaku AFC–7R diffractometer	*R*_int_ = 0.052
ω scans	θ_max_ = 27.8°, θ_min_ = 3.1°
Absorption correction: ψ scan (North et al., 1968)	*h* = −4→2
*T*_min_ = 0.574, *T*_max_ = 0.934	*k* = −7→7
5075 measured reflections	*l* = −50→50
2089 independent reflections	3 standard reflections every 150 reflections
1747 reflections with *F*^2^ > 2.0σ(*F*^2^)	intensity decay: 0.6%

### Refinement

**Table:**

Refinement on *F*^2^	Secondary atom site location: difference Fourier map
*R*[*F*^2^ > 2σ(*F*^2^)] = 0.034	Hydrogen site location: inferred from neighbouring sites
*wR*(*F*^2^) = 0.098	H-atom parameters constrained
*S* = 1.04	*w* = 1/[σ^2^(*F*_o_^2^) + (0.0487*P*)^2^ + 0.5553*P*] where *P* = (*F*_o_^2^ + 2*F*_c_^2^)/3
2089 reflections	(Δ/σ)_max_ < 0.001
136 parameters	Δρ_max_ = 0.29 e Å^−^^3^
0 restraints	Δρ_min_ = −0.36 e Å^−^^3^
Primary atom site location: structure-invariant direct methods	

### Special details

**Table:**

Geometry. All e.s.d.'s (except the e.s.d. in the dihedral angle between two l.s. planes) are estimated using the full covariance matrix. The cell e.s.d.'s are taken into account individually in the estimation of e.s.d.'s in distances, angles and torsion angles; correlations between e.s.d.'s in cell parameters are only used when they are defined by crystal symmetry. An approximate (isotropic) treatment of cell e.s.d.'s is used for estimating e.s.d.'s involving l.s. planes.
Refinement. Refinement was performed using all reflections. The weighted *R*-factor (*wR*) and goodness of fit (*S*) are based on *F*^2^. *R*-factor (gt) are based on *F*. The threshold expression of *F*^2^ > 2.0 *σ*(*F*^2^) is used only for calculating *R*-factor (gt).

### Fractional atomic coordinates and isotropic or equivalent isotropic displacement parameters (Å^2^)

**Table:**

	*x*	*y*	*z*	*U*_iso_*/*U*_eq_	
Cl1	0.55004 (14)	0.47912 (8)	0.27821 (2)	0.02586 (15)	
Cl2	0.92228 (14)	0.92474 (8)	0.29767 (2)	0.02698 (15)	
O1	0.8830 (4)	0.6908 (2)	0.41933 (3)	0.0230 (3)	
O2	0.3678 (4)	0.1171 (2)	0.40111 (3)	0.0274 (3)	
O3	0.7039 (5)	0.2474 (3)	0.49732 (4)	0.0373 (4)	
C1	0.8118 (6)	0.5474 (3)	0.44408 (5)	0.0224 (4)	
H1	0.8796	0.5864	0.4666	0.027*	
C2	0.6523 (5)	0.3530 (3)	0.43968 (4)	0.0208 (4)	
C3	0.5328 (5)	0.2856 (3)	0.40621 (5)	0.0199 (4)	
C4	0.5555 (5)	0.3935 (3)	0.34486 (4)	0.0192 (4)	
H2	0.4430	0.2604	0.3389	0.023*	
C5	0.6442 (5)	0.5401 (3)	0.31980 (5)	0.0197 (4)	
C6	0.8083 (5)	0.7381 (3)	0.32843 (4)	0.0189 (4)	
C7	0.8846 (5)	0.7862 (3)	0.36175 (5)	0.0199 (4)	
H3	0.9962	0.9196	0.3678	0.024*	
C8	0.6295 (5)	0.4389 (3)	0.37891 (4)	0.0180 (4)	
C9	0.7948 (5)	0.6354 (3)	0.38648 (4)	0.0188 (4)	
C10	0.5952 (6)	0.2085 (3)	0.46908 (5)	0.0278 (5)	
H4	0.4657	0.0777	0.4655	0.033*	

### Atomic displacement parameters (Å^2^)

**Table:**

	*U*^11^	*U*^22^	*U*^33^	*U*^12^	*U*^13^	*U*^23^
Cl1	0.0352 (3)	0.0278 (3)	0.0144 (2)	−0.0004 (2)	−0.00603 (19)	−0.00328 (17)
Cl2	0.0343 (3)	0.0257 (3)	0.0209 (2)	−0.0016 (2)	−0.0012 (2)	0.00378 (18)
O1	0.0335 (8)	0.0206 (7)	0.0148 (6)	−0.0087 (6)	−0.0050 (5)	−0.0014 (5)
O2	0.0360 (9)	0.0226 (7)	0.0234 (7)	−0.0121 (6)	−0.0061 (6)	−0.0005 (6)
O3	0.0558 (11)	0.0357 (9)	0.0200 (7)	−0.0143 (8)	−0.0102 (7)	0.0052 (6)
C1	0.0285 (11)	0.0233 (9)	0.0153 (8)	−0.0030 (8)	−0.0021 (7)	−0.0011 (7)
C2	0.0253 (10)	0.0205 (9)	0.0166 (9)	−0.0041 (8)	−0.0024 (7)	−0.0002 (7)
C3	0.0229 (10)	0.0192 (9)	0.0175 (9)	−0.0013 (7)	−0.0018 (7)	−0.0019 (7)
C4	0.0224 (10)	0.0177 (8)	0.0174 (8)	−0.0008 (7)	−0.0033 (7)	−0.0037 (7)
C5	0.0223 (9)	0.0217 (9)	0.0149 (8)	0.0018 (7)	−0.0029 (7)	−0.0034 (7)
C6	0.0219 (10)	0.0186 (9)	0.0161 (8)	0.0004 (7)	−0.0016 (7)	0.0021 (7)
C7	0.0222 (10)	0.0175 (8)	0.0197 (9)	−0.0029 (7)	−0.0023 (7)	−0.0020 (7)
C8	0.0209 (9)	0.0170 (8)	0.0161 (8)	−0.0013 (7)	−0.0029 (7)	−0.0017 (6)
C9	0.0225 (9)	0.0193 (8)	0.0146 (8)	−0.0012 (7)	−0.0035 (7)	−0.0034 (7)
C10	0.0370 (12)	0.0265 (10)	0.0199 (9)	−0.0081 (9)	−0.0032 (8)	0.0023 (8)

### Geometric parameters (Å, º)

**Table:**

Cl1—C5	1.7148 (19)	C3—C8	1.480 (3)
Cl2—C6	1.7275 (19)	C4—C5	1.381 (3)
O1—C1	1.344 (2)	C4—C8	1.396 (2)
O1—C9	1.376 (2)	C4—H2	0.9500
O2—C3	1.223 (2)	C5—C6	1.405 (3)
O3—C10	1.206 (2)	C6—C7	1.374 (2)
C1—C2	1.348 (3)	C7—C9	1.391 (3)
C1—H1	0.9500	C7—H3	0.9500
C2—C3	1.450 (2)	C8—C9	1.390 (3)
C2—C10	1.478 (3)	C10—H4	0.9500
			
C1—O1—C9	118.23 (15)	C7—C6—C5	120.28 (17)
O1—C1—C2	125.47 (16)	C7—C6—Cl2	118.54 (15)
O1—C1—H1	117.3	C5—C6—Cl2	121.18 (14)
C2—C1—H1	117.3	C6—C7—C9	118.52 (17)
C1—C2—C3	120.25 (17)	C6—C7—H3	120.7
C1—C2—C10	120.05 (17)	C9—C7—H3	120.7
C3—C2—C10	119.69 (17)	C9—C8—C4	117.65 (17)
O2—C3—C2	122.92 (17)	C9—C8—C3	120.72 (16)
O2—C3—C8	123.27 (16)	C4—C8—C3	121.63 (16)
C2—C3—C8	113.81 (16)	O1—C9—C8	121.32 (16)
C5—C4—C8	120.70 (17)	O1—C9—C7	115.91 (16)
C5—C4—H2	119.7	C8—C9—C7	122.76 (16)
C8—C4—H2	119.7	O3—C10—C2	123.64 (19)
C4—C5—C6	120.10 (16)	O3—C10—H4	118.2
C4—C5—Cl1	119.51 (14)	C2—C10—H4	118.2
C6—C5—Cl1	120.40 (15)		
			
C9—O1—C1—C2	−1.5 (3)	C5—C4—C8—C3	179.21 (18)
O1—C1—C2—C3	−1.7 (3)	O2—C3—C8—C9	175.37 (19)
O1—C1—C2—C10	178.9 (2)	C2—C3—C8—C9	−4.8 (3)
C1—C2—C3—O2	−175.48 (19)	O2—C3—C8—C4	−3.9 (3)
C10—C2—C3—O2	3.9 (3)	C2—C3—C8—C4	175.93 (17)
C1—C2—C3—C8	4.7 (3)	C1—O1—C9—C8	1.4 (3)
C10—C2—C3—C8	−175.96 (18)	C1—O1—C9—C7	−177.98 (17)
C8—C4—C5—C6	−0.5 (3)	C4—C8—C9—O1	−178.76 (17)
C8—C4—C5—Cl1	179.93 (15)	C3—C8—C9—O1	1.9 (3)
C4—C5—C6—C7	0.6 (3)	C4—C8—C9—C7	0.6 (3)
Cl1—C5—C6—C7	−179.76 (15)	C3—C8—C9—C7	−178.76 (18)
C4—C5—C6—Cl2	−179.99 (15)	C6—C7—C9—O1	178.95 (17)
Cl1—C5—C6—Cl2	−0.4 (2)	C6—C7—C9—C8	−0.4 (3)
C5—C6—C7—C9	−0.2 (3)	C1—C2—C10—O3	−4.9 (4)
Cl2—C6—C7—C9	−179.59 (15)	C3—C2—C10—O3	175.8 (2)
C5—C4—C8—C9	−0.1 (3)		

### Hydrogen-bond geometry (Å, º)

**Table:**

*D*—H···*A*	*D*—H	H···*A*	*D*···*A*	*D*—H···*A*
C1—H1···O3^i^	0.95	2.34	3.187 (3)	148 (1)
C7—H3···O2^ii^	0.95	2.26	3.129 (2)	151 (1)

Symmetry codes: (i) −*x*+2, −*y*+1, −*z*+1; (ii) *x*+1, *y*+1, *z*.
