# Supplementary figures and images for: Crystal structure of 6,7-di­chloro-4-oxo-4H-chromene-3-carbaldehyde
Source: Acta Crystallogr E Crystallogr Commun. 2015 Aug 12;71(Pt 9):o652–3. doi: 10.1107/S2056989015014644 (PMC4555387; doi:10.1107/S2056989015014644)

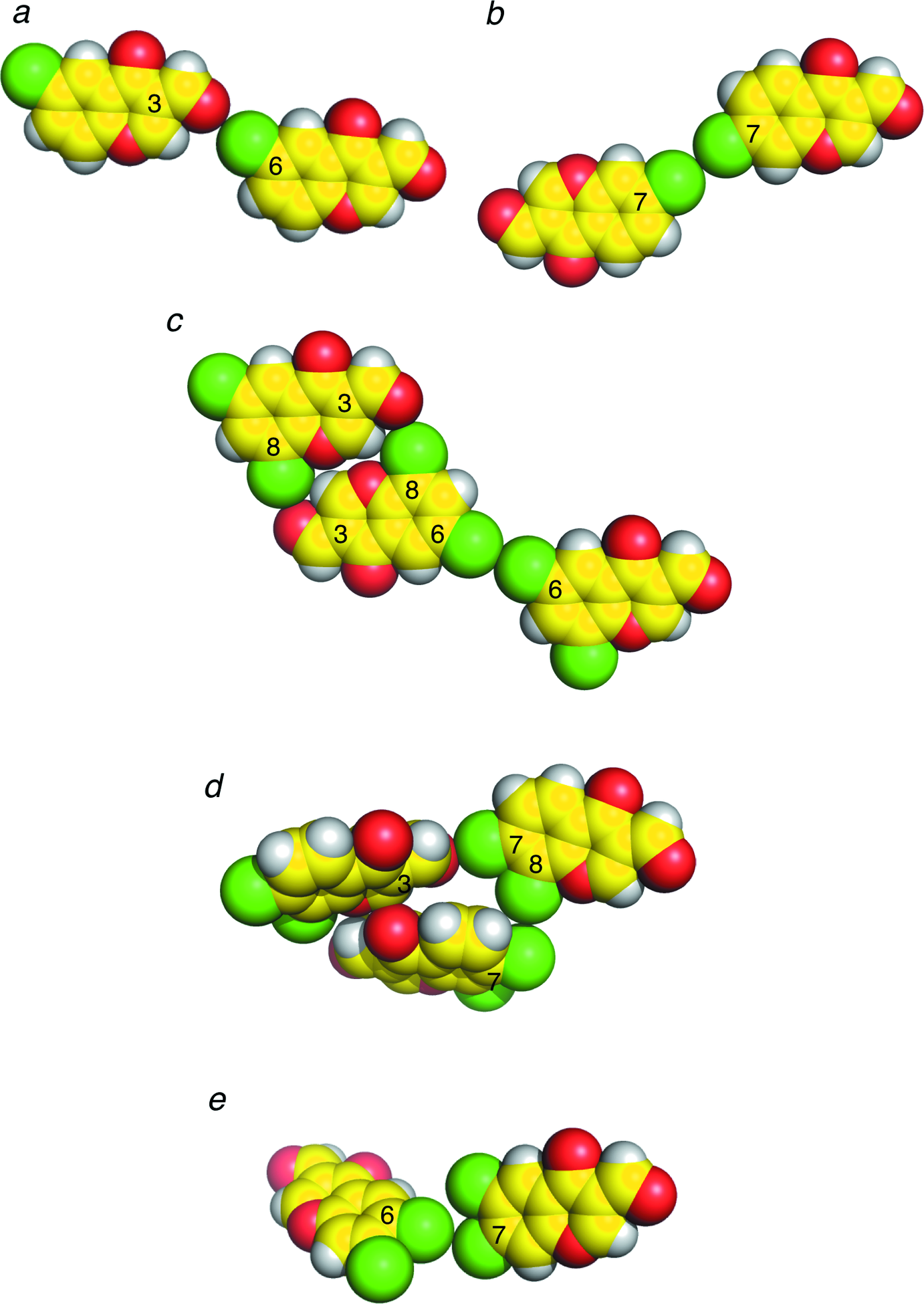

Supplement: Supplementary file 4 [file e-71-0o652-fig1.tif]

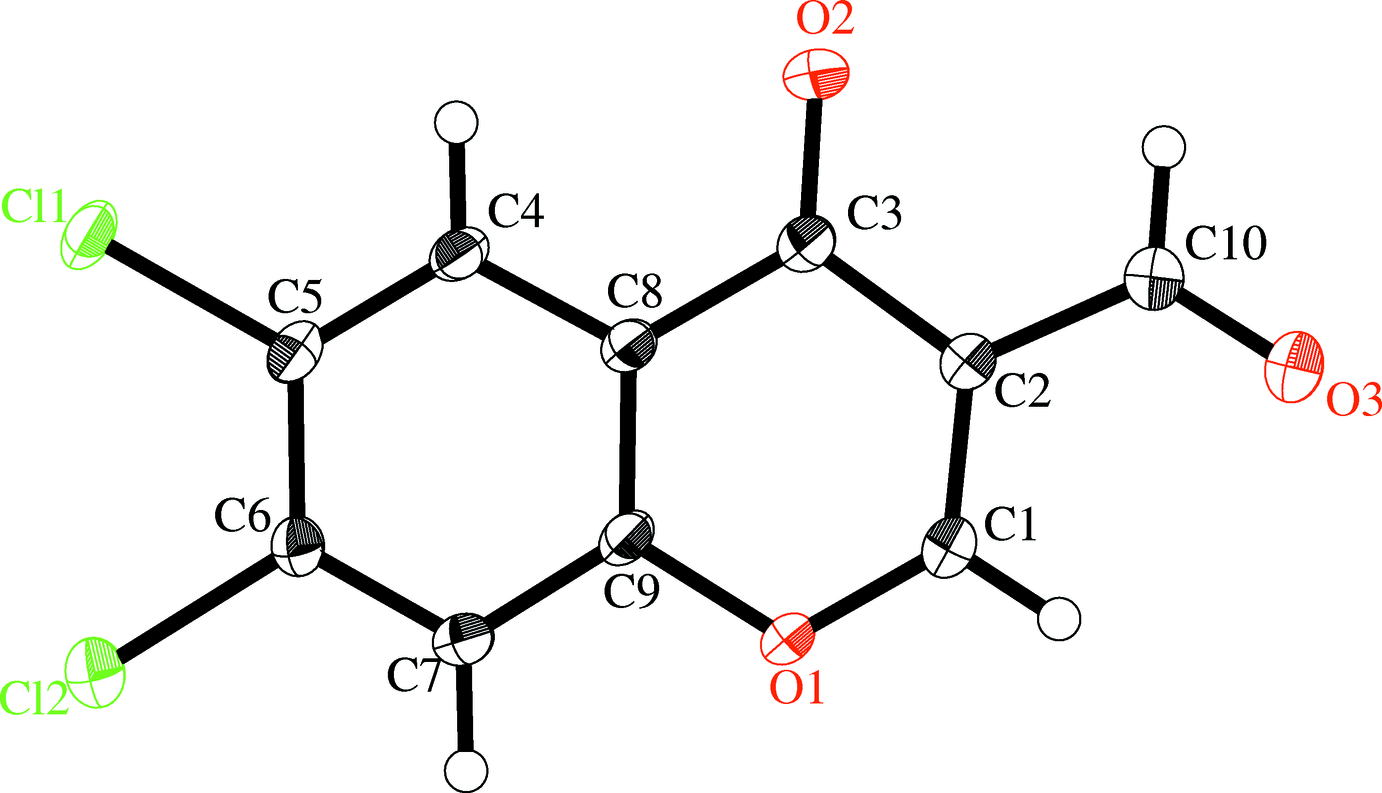

Supplement: Supplementary file 5 [file e-71-0o652-fig2.tif]

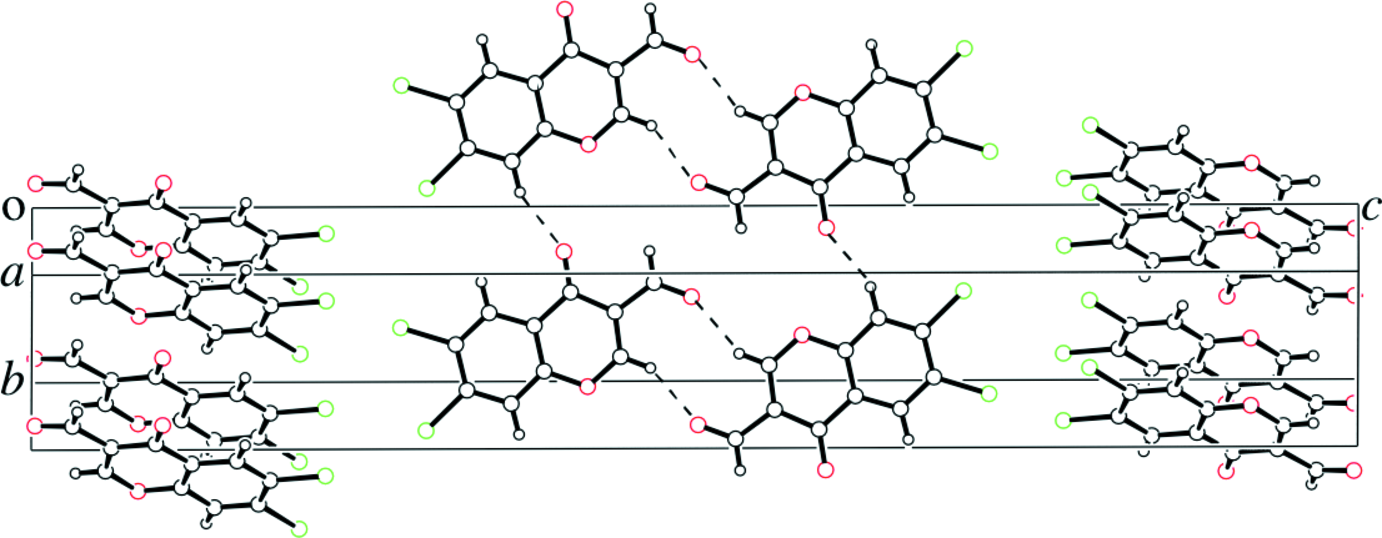

Supplement: Supplementary file 6 [file e-71-0o652-fig3.tif]
